# Finite Element Modeling of Bond Behavior of FRP and Steel Plates

**DOI:** 10.3390/ma14040757

**Published:** 2021-02-05

**Authors:** Elena Ciampa, Francesca Ceroni, Maria Rosaria Pecce

**Affiliations:** 1 Department of Engineering, University of Sannio, Piazza Roma, 82100 Benevento, Italy; pecce@unisannio.it; 2 Department of Engineering, University of Napoli Parthenope, Centro Direzionale, 80143 Naples, Italy; francesca.ceroni@uniparthenope.it

**Keywords:** FRP, steel plate, concrete, externally bonded strengthening, bond behavior, 3D finite element model

## Abstract

Strengthening systems for existing reinforced concrete (RC) structures are increasingly needed due to several problems such as degradation of materials over the time, underdesign, serviceability or seismic upgrading, or new code requirements. In the last decades, strengthening by fibers composite materials applied with various techniques (FRP, FRCM, NSM) were largely investigated and theoretical formulations have been introduced in national and international design guidelines. Although they are an excellent strengthening solution, steel plates may represent still a valid traditional alternative, due to low costs, ductile stress-strain behavior, simple and fast mounting with possibility of reusing the material. Guidelines for a correct design are still lack and, therefore, detailed models and design formulas are needed. In this paper, the bond behavior at the plate-concrete interface, which plays a key role for the effectiveness of the strengthening system, is analyzed by means of 3D finite element models calibrated on experimental results available in literature. Parametric analyses were carried out by changing some meaningful parameters.

## 1. Introduction

In the last decades, the exponential growth of strengthening interventions for deficient reinforced concrete (RC) structures has led to a rapid development of innovative materials and techniques. Externally bonded fiber reinforced polymer (FRP) plates and sheets have been increasingly used to replace steel plates for strengthening RC structures thanks to light weight, high strength, easy application and availability of different sizes and dimensions. On the other hand, FRP materials have a brittle stress-strain behavior, which may also limit the overall ductility of the strengthened elements. Moreover, premature loss of bond at the concrete/glued plate interface does not allow to fulfil the high performance of FRP materials. In fact, since the interfacial bond behavior plays a key role in the performance and the durability of the strengthened element, several experimental, analytical and numerical studies about bond behavior and debonding failure modes have been performed over the years. Debonding has been widely studied for FRP materials externally bonded to concrete substrates [1,2,3,4], while less studies for glued steel plates are available. Thus, most experimental results available in the scientific literature concern bond tests on FRP materials glued to concrete elements, while only few tests, not recent, were carried out on steel plate glued to concrete [5,6,7]. Externally bonded or bolted steel plates represented, indeed, the first example of external strengthening technique for existing RC elements and they were supplanted by FRP materials in the 1990s. However, they still may represent a significant strengthening alternative thanks to the relatively lower prices, ductile stress-strain constitutive behavior of steel, simple and fast mounting with possibility of removing and eventually reusing the material.

The purpose of this study is to investigate the bond behavior and the bond strength of steel plates externally bonded to concrete elements by means of a refined three-dimensional (3D) finite element (FE) model, implemented in the program MIDAS FEA NX [8]. The model was firstly validated by means of comparisons with experimental results of bond tests carried out in [9] on carbon FRP plate bonded to concrete elements. In addition, a comparison between the results (i.e., debonding load and effective transfer length) provided by the 3D FE model when the interface is modeled by means of a non-linear bond law or when a real epoxy layer is simulated is presented too.

Finally, after the validation of the FE models, several parametric analyses for the case of external reinforcement made of steel plates have been carried out by changing some meaningful parameters (thickness, width and bonded length of the plate, compressive strength of the concrete). The results have been also discussed by comparing them with theoretical values provided by literature strength models.

The study represents the first step of a wider research involving both experimental tests and numerical analyses on steel plates externally glued on concrete elements and aimed to verify the reliability of the actual approaches proposed for FRP plates for the steel ones too. It is worth noting, indeed, that for FRP externally reinforcements, the formulations and the design factors currently suggested in national and international guidelines are based on a wide database of experimental tests, while a limited number of results are available for steel plates.

## 2. Literature Review

**Figure 1:**
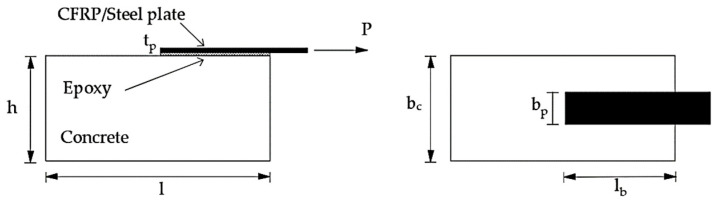
Scheme of single shear bond test.

Debonding phenomena in externally strengthened concrete elements have been extensively studied in case of FRP plates, while few results are available for steel plates. A great deal of bond tests for FRP-concrete system have been, indeed, carried out in the past, using several set-ups including single shear test [4,10,11] as shown in Figure 1, double shear test [12] or modified beam-test [13]. However, codified procedure for performing bond tests are still lacking, even if in [2] the analysis of a wide database of results coming from bond tests on FRP/concrete systems and realized according to different set-ups showed very scattered results depending on the testing procedures.

Bond tests are mainly aimed to investigate the bond strength, the effective bond length and the bond shear stress-slip relationships. All these topics depend on both geometrical and mechanical properties of the strengthening system and of the substrate. The bond strength is the maximum tensile load or stress applicable in the external reinforcement to attain a “bond” failure, usually indicated in literature as “debonding”, i.e., a detachment of the reinforcement from the substrate. The effective bond length is the length beyond which a further extension of the reinforcement cannot increase the load capacity of the strengthened element [14,15,16]. However, it is worth noting that bond lengths longer than the effective one should be favorable, since they may improve the ductility of the debonding process [16], even if the failure load does not increase anymore. The bond shear-stress law is indicative of the quality of the bond behavior and allows defining both the efficiency of the connection between the substrate and the external reinforcement under serviceability load conditions and the bond strength, i.e., the efficiency of the glued joint at ultimate load conditions.

Since the “debonding” often occurs with detachment of a thin layer beneath the concrete surface, concrete strength and surface condition affect significantly the bond strength [17,18]. However, the bond strength is affected also by the geometrical and mechanical properties of both the bonded plate and the adhesive [14], i.e., bond length and axial stiffness of the plate, plate-to-concrete width ratio, axial stiffness and tensile strength of the adhesive.

Several theoretical formulations have been proposed in the past to evaluate the bond strength and the effective bond length for both FRP plates or sheets and steel plates [13,14,19,20,21]. These formulations contemplate the aforementioned parameters and some of them introduce the fracture energy, G_f_, which represents the mode II energy released by the concrete/plate joint during the debonding process and is related to its maximum load capacity.

Analysis of literature evidenced that existing bond strength models may be classified into three categories as suggested in [16]: (1) empirical models based directly on the regression of experimental data, (2) models based on fracture energy, and (3) design proposals that make use of some simple assumptions.

In [19], a series of bond tests on carbon FRP reinforcements bonded on concrete elements were reported and a modified form of the Holzenkämpfer’s model [22], based on fracture mechanism approach, to predict ultimate load referred to steel plate was proposed too. 

In [23], the model of Chen and Teng [16] is modified by means of a numerical factor aimed to take into account the intermediate crack debonding in RC beams and slabs. In [21], an expression for the maximum transferable load in case of bond length longer than effective bond length is proposed too. Brosens and Gemert, in 1999 [24], recognized the effect of the adhesive layer on the bond law and, thus, on the maximum shear stress, the corresponding slip and the ultimate slip as well as on the effective length. Ueda et al., in 2003 [25], considered the shear modulus, G_a_, and the thickness, t_a_, of the adhesive directly in the expression of the maximum shear stress. Dai et al., in 2005 [18], proved that the debonding load increases when the stiffness of the reinforcement increases, but especially when the shear stiffness of the adhesive reduces. Gonzalez et al., in 2012 [26], found that adhesive thickness has no significant impact on low-strength concrete, but it may have a little effect on higher strength substrates. The studies demonstrated, indeed, that in higher strength concrete, failure occurred at the concrete-adhesive interface, which was the weakest component, and, in such a case, the adhesive plays a more significant role, since a greater adhesive thickness would favor a more homogeneous shear stress distribution and increase the ultimate load. However, it is worth noting that the effect of the geometrical and mechanical properties of the adhesive layer has been barely investigated.

In this paper, two strength models have been considered for both FRP and steel plates, for calculating the effective length L_e_ and the maximum tensile load in the reinforcement, P_u_, i.e., the debonding load. The first strength model (Equations (1)–(3)) is the one proposed in the recent *fib* Bulletin 90 [27], which is specific for externally bonded FRP reinforcement for concrete structures, while the second one is the Chen and Teng’s model (Equations (4)–(6)) [16], which refers to both externally bonded FRP and steel plates. Since debonding generally occurs in the concrete, effective length and bond strength depend on mechanical properties of the concrete. In particular, the *fib* Bulletin 90 [27] approach considers the fracture energy as main parameter, unlike Chen and Teng’s model [16], which refers directly to the compressive strength of concrete and to the properties of the reinforcement. Moreover, the *fib* Bulletin 90 provides both mean and 5% percentile (i.e., characteristic values) predictions for L_e_ and P_u_, since it is based on a wide database of experimental results of bond tests on FRP materials applied to concrete elements.

(1) $$L_{e}=k_{l}\frac{\pi }{k_{b}}\sqrt{\frac{E_{p}\cdot t_{p}}{{8\cdot f}_{\mathrm{cm}}^{2/3}}}$$

where $k_{l}$ = 1 for mean value and $k_{l}$ = 1.5 for 5% percentile value.

(2) $$\begin{array}{l} k_{b}=\sqrt{\frac{\left(2-\frac{b_{p}}{b_{c}}\right)}{1+\frac{b_{p}}{b_{c}}}\geq 1}\\ \beta_{L}=\{\begin{array}{ll} 1 & \;\mathrm{if}\;l_{b}\geq L_{e}\\ \frac{l_{b}}{L_{e}}\left(2-\frac{l_{b}}{L_{e}}\right)\;<\;1 & \;\mathrm{if}\;l_{b}{\;<\;L}_{e} \end{array}\\ G_{f}=k^{2}k_{b}^{2}f_{\mathrm{cm}}^{2/3} \end{array}$$

where k = 0.25 for mean value and k = 0.17 for 5% percentile value.

(3) $$P_{u}=\beta_{L}b_{p}\sqrt{{2E}_{p}t_{p}G_{f}}$$

(4) $$L_{e}=\sqrt{\frac{E_{p}{\cdot t}_{p}}{\sqrt{f_{\mathrm{cm}}}}}$$

(5) $$\begin{array}{l} k_{b}=\sqrt{\frac{\left(2-\frac{b_{p}}{b_{c}}\right)}{1+\frac{b_{p}}{b_{c}}}}\\ \beta_{L}=\{\begin{array}{ll} 1 & \mathrm{if}\;l_{b}\;\geq \;L_{e}\\ \sin\left[\frac{{\pi \cdot l}_{b}}{{2\cdot L}_{e}}\right] & \mathrm{if}\;l_{b}\;<\;L_{e} \end{array} \end{array}$$

(6) $$P_{u}=0.427\cdot k_{b}\cdot \beta_{L}\sqrt{f_{c}}\cdot b_{p}\cdot L_{e}$$

In both formulations, E_p_, t_p_, b_p_ and l_b_ are the Young modulus, thickness, width and bonded length of the strengthening plate, while b_c_ and f_cm_ are the width and the mean compressive strength of the concrete. k_b_ is the shape factor aimed to take into account the actual volume of concrete involved in the debonding mechanism and not only the one under the bonded area, while β_L_ is a reductive factor of the debonding load in case of bonded length lower than the effective one.

Note that in *fib* Bulletin 90 [27] the shape factor k_b_ is enshrined also in the evaluation of the effective length that, thus, increases with the width of the plate.

## 3. Modeling Approaches for Bonded Interface

The debonding process in strengthened RC elements with externally bonded plates can be modeled involving one-dimensional, two-dimensional or three-dimensional approaches.

The simplest approach is the one-dimensional where the adhesive can be simulated by means of horizontal and vertical independent springs [28]. In this approach, the axial and flexural stiffness of the concrete block can be considered infinite compared to that of the glued plate and, thus, it is not necessary to introduce the mechanical and geometric characteristics of the concrete. Clearly, the fault of this approach is the loss of knowledge about the effects induced in the concrete by the bond stress transfer. Moreover, in this approach the differences in the bond stress distributions along the concrete–adhesive and the adhesive-plate interfaces are neglected.

Two-dimensional (2D) and three-dimensional (3D) approaches, usually implemented in finite element (FE) models, allow to take into account the variation of stresses along the thickness of the adhesive. Two-dimensional models allow calibrating more reliably than the one-dimensional the main parameters of the bond law by means of comparisons with experimental results. However, in order to take into account also the transversal effects for a more realistic and comprehensive study of the debonding process, the three-dimensional models are the most suitable ones [29]. It is worth noting that the 3D models are also the most complex to implement, with a lot of tricky aspects regarding the correct choice of the parameters for defining damage laws and non-linear behavior of both materials and interfaces.

**Figure 2:**
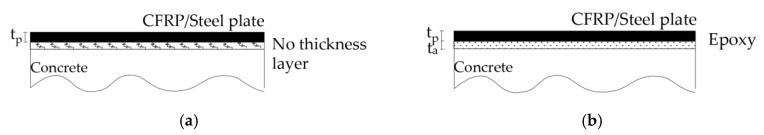
Modeling of adhesive layer with: (**a**) non-linear springs; (**b**) real epoxy layer.

In FE models, there are generally two possibilities for simulating the debonding process in externally strengthened RC elements. The first one considers a discrete or a smeared crack model for the concrete, while the plate–concrete interface is modeled by means of zero-thickness interfacial elements (nonlinear springs, Figure 2a), which are characterized by nonlinear shear stress-slip laws only along the direction of the bonded interfaces [30,31]. Usually, along the direction orthogonal to the bonded surface, a very high stiffness is, indeed, assumed [32].

The second approach simulates the real layer of adhesive with its thickness (Figure 2b) and, thus, the failures in the materials (substrate, adhesive, reinforcement) are directly simulated by specific constitutive models [33].

## 4. The FE 3D model

In order to simulate a single push-pull test on FRP or steel plates externally bonded to a concrete element, a non-linear three-dimensional (3D) FE model was developed using the software Midas FEA NX [8]. The FE model was aimed to investigate both the local bond behavior at the concrete/reinforcement interface in terms of strain distribution in the external reinforcement and the global behavior in term of debonding strength. Some experimental tests available in literature and related to the use of CFRP plates [9] bonded to concrete elements were used to calibrate the FE model. Successively, the FE model was used for carrying out parametric analyses with reference to steel plates bonded on concrete elements.

**Figure 3:**
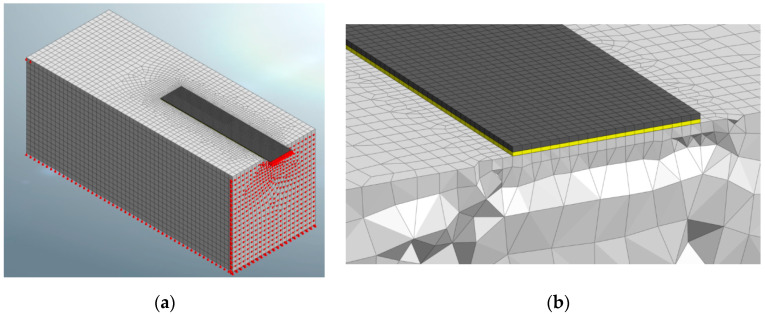
The 3D model: (**a**) global view; (**b**) detail of the elements shape.

A FE 3D configuration of the specimen simulating the push–pull test was implemented in the software (Figure 3a). Since the push–pull test provides a compression applied to the specimen, usually by means of stiff contrasting elements, and a tension force applied to the external reinforcement [34], fixed constraints were placed on the front of the concrete block to prevent horizontal displacements and simulate the presence of the contrasting elements. Constraints were placed also on the bottom side of the block and along the back edge of the upper face of the block in order to prevent vertical movements. The loaded end of the reinforcement is also restrained in order to allow only the longitudinal displacement, as in the experimental tests [9] where it is provided by a suitable clamping device.

Solid elements with hybrid geometry, as shown in Figure 3b were implemented. The greatest part of the block was made of hexahedron elements, while some pyramidal and tetrahedron elements were combined to ensure the node connections with the adhesive and the plate [8].

The mesh size of the 3D model, especially at the interface of the plates, can influence the results; therefore, it was established considering the smaller dimension of the bonded area that is represented by the plate width. The width of the plates considered in the analyses varies in the range of 10−50 mm. The optimization of the mesh along the width and length of the plate was carried out varying the element sides at the interface between 7 mm and 1.5 mm. For the specimens with a plate width of 30 mm and 50 mm, the dimensions of 2.5 mm for the plate and adhesive meshes give the best solution because the further reduction to 1.5 mm allows a variation of only 2% of the ultimate load, but leads to a great increment of the calculation time too. For the specimens with a plate width of 10 mm, the dimensions of 1.5 mm for the plate and adhesive has been adopted since the variation in the results from 2.5 mm to 1.5 mm was of about 8%. The thickness of both plate and adhesive is not divided in further elements when the mesh size is higher or close to the real thickness (i.e., mesh 2.5 mm respect to plate thickness of 2 or 3 mm). Conversely, the thickness is also divided when the mesh is much lower than the thickness (i.e., the mesh 1.5 mm in case of plate thickness of 3 mm gives two elements along the thickness). The number of finite elements is, thus, different according to the different dimensions of the analyzed plates, but a maximum number of approximately 56000 elements was used.

All the analyses were performed under displacement control through the incremental application of a monotonic displacement of 0.01 mm at the loaded end of the plate.

Two approaches were considered for modeling the connection between the concrete and the external plate: (a) use non-linear interface elements, and (b) simulation of the epoxy layer with its thickness. A comparison between the results provided by these two approaches is then proposed.

### 4.1. 3D FE Model with Nonlinear Interface Elements (IE Approach)

**Figure 4:**
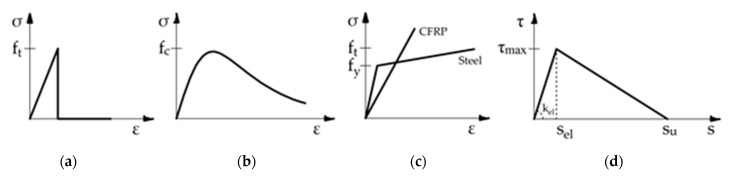
Constitutive laws for materials and bond behavior: (**a**) Tensile stress-strain relationship for concrete; (**b**) stress-strain relationship for concrete in compression; (**c**) Tensile stress-strain relationships for CFRP and steel plates; (**d**) Bilinear interfacial bond law.

When non-linear interface elements are used, a zero thickness layer, which directly connects the external reinforcement and the substrate, is introduced (see Figure 2a) and the smeared crack model for concrete is the usual choice. Such a modeling strategy, labeled in the following as “IE approach”, leads the debonding process develops with distributed cracks. The response of concrete in tension is assumed linear elastic with a brittle crisis when the fracture surface is reached (Figure 4a). The tensile strength of concrete clearly plays a key role in the fracture mechanism. The constitutive model suggested by Thorenfeldt [35], characterized by a quite linear law in the elastic range followed by a parabolic trend and by a nonlinear softening branch (Figure 4b), was used to describe the compressive behavior of concrete. It is worth noting that the failure mechanics in the compression of the concrete is usually not dominant during the bond failure.

The CFRP and the steel plates were modeled as orthotropic and isotropic materials, respectively. The FRP plate exhibits a linear elastic behavior up to failure in tension (Figure 4c), while no contribute in compression is considered. Conversely, the steel plate has a typical elastic-plastic behavior both in tension and compression with a hardening branch after yielding. The Von Mises criterion with an associated isotropic hardening was assumed.

The bond law used for the interface elements is a simplified bi-linear law as shown in Figure 4d where τ_max_ is the maximum shear stress (shear bond strength), s_el_ is the corresponding slip, and s_u_ is the ultimate slip after which no further shear stresses can be transferred through the interface.

### 4.2. 3D FE Model with Adhesive Layer (AL Approach)

When the connection between the external reinforcement and the substrate is modeled considering explicitly the presence of the adhesive (Figure 2b), the latter is simulated as a further intermediate layer equipped with its own thickness and with a linear elastic isotropic behavior; this simple model for the adhesive can be assumed because the concrete failure occurs when it is still in the elastic field. Thus, for this approach, labelled in the following as “AL approach”, no bond law is assumed along both interfaces, i.e., the substrate-adhesive and the adhesive-reinforcement ones.

**Figure 5:**
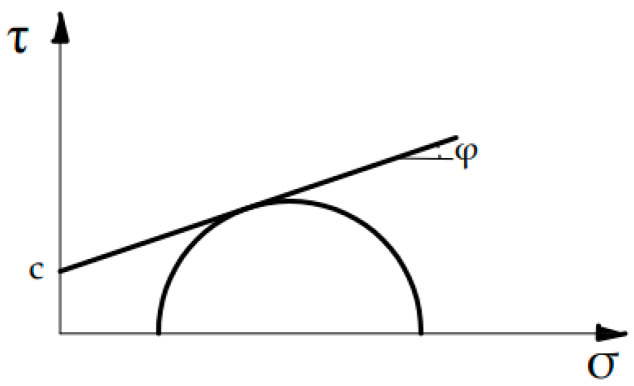
Mohr-Coulomb stress-strain relationship for concrete in FE model with epoxy layer.

The constitutive laws of steel and FRP plates are the same shown in Figure 4c. Conversely, the concrete was modeled considering a Mohr–Coulomb criterion (Figure 5) with a tension cut-off defined by the Rankine model. It requires that the interface cohesion, c, the friction angle, φ, and the tensile strength, f_t_, are assigned. These parameters have been calibrated basing on the experimental data of [9] and the experimental study of [36] was taken into account too.

## 5. Analysis of Bond Behavior by Means of FE Models 

### 5.1. Validation of the FE Models by Means of Comparisons with Experimental Results

The concrete blocks used in the bond test in [9] had a square cross section with side 200 mm and a length of 500 mm. Carbon FRP (CFRP) plates with thickness of 1.4 mm were glued on concrete specimens by means of an epoxy adhesive. The experimental parameters variable in the experimental campaign were the compressive strength of concrete, f_c_, the width, b_p_, and the bonded length, l_b_ of the CFRP reinforcement. 

Firstly, the two approaches implemented in the 3D FE model, i.e., the IE and the AL ones previously introduced, have been compared with the experimental results of [9] in terms of both maximum load and strain distributions along the plate. Such a comparison allowed to assess the parameters of the bond law necessary in the IE approach and the parameters of the Mohr-Coulomb criterion necessary for the AL one.

In the IE approach, a simplified bilinear bond-slip law, τ–s, (Figure 4d) according to the suggestion of Woo and Lee [9], was considered. For specimens with concrete C30, the maximum bond stress and the corresponding slip are τ_max_ = 4.5 MPa and s_el_ = 0.06 mm, while the ultimate slip is s_u_ = 0.30 mm. For specimens with concrete C50, different values are considered, i.e., τ_max_ = 7.0 MPa and the corresponding slip s_el_ = 0.09 mm, while the ultimate slip is s_u_ = 0.30 mm.

In the AL approach, the following data have been assumed:

1. for the concrete with characteristic compressive strength of 30 and 50 MPa [9], the mean value of the compression strength, f_cm_, has been assumed equal to 38 MPa and 58 MPa, according to Eurocode 2 [37];
2. the tensile strength and the elastic modulus of concrete have been evaluated from the compression strength according to formulations provided by Eurocode 2 [37];
3. the value of the cohesion, *c*, of the Mohr Coulomb criterion is assumed 1.8 and 2.6 MPa for the two concretes with f_cm_ = 38 MPa and 58 MPa, respectively, while the friction angle, ϕ, is assumed 31° as provided by Lelović et al. [36] for both of them;
4. the adhesive thickness was assumed 1 mm and 3 mm and the shear modulus G_a_ was calibrated basing on the experimental distribution of the strain along the strengthening plate. It resulted equal to 0.18 GPa and, however, it was also verified that it provides a low influence on the ultimate load.

A summary of the materials properties used in FE models are listed in Table 1.

Two specimens tested by [9] were simulated by both the FE approaches. The specimens are characterized by two different compressive strengths of concrete, same bonded length (l_b_ = 250 mm) and widths (b_p_ = 50 mm). The main geometrical data of these specimens are listed in Table 2.

In Table 2, the maximum experimental loads, P_u,exp_, of the three specimens are compared with the numerical values achieved by the two FE models, P_u_,_num_, and with the theoretical values, P_u,th_, provided by the strength models represented by Equation (3) (mean value) and Equation (6).

In Table 2 the differences between the experimental values and the theoretical and numerical ones are listed too in terms of ratios P_u,exp_/P_u,th_, P_u,exp_/P_u_,_num_ for both AL and IE approaches, and P_u_,_num_^AL^/P_u,th_. It is worth noting that the experimental values are different from the theoretical ones in the range of −14% and +6%. Moreover, the difference between the experimental and the numerical values is lower than 9%. Finally, the differences between the numerical values provided by the AL approach and the theoretical ones are in the range −14% and +11%. Equation (3) generally provides higher values than Equation (6).

**Figure 6:**
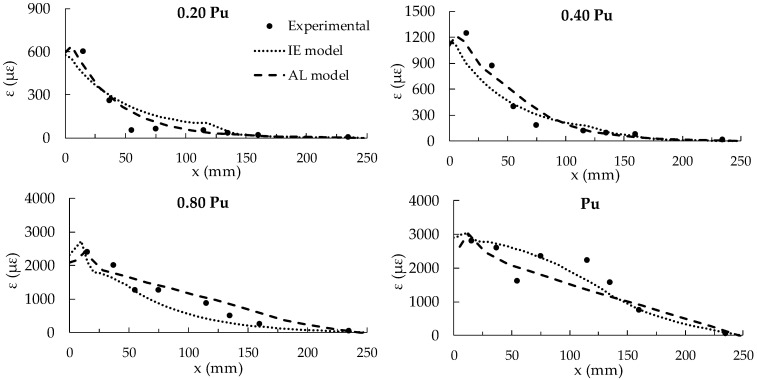
Experimental vs. numerical strain distributions provided by IE and AL FE models for specimen C30_1.4_50_250 tested in [9].

In Figure 6, for the specimen C30_1.4_50_250, the experimental strain distributions along the CFRP plate, assuming as origin of the axis x the edge of the bonded length at the loaded side, are compared with the numerical results provided by both the 3D FE models, IE and AL, at four load levels, i.e., 20%, 40%, 80% and 100% of P_u_. The graphs show that the agreement between the experimental and the numerical results is not very satisfactory for the lower load levels, while improves at the higher ones. In particular, it can be observed that the two approaches give very similar results for the lower load levels (0.2 and 0.4 P_u_), which correspond to the elastic branch of the bond law for both approaches. At higher load levels, the AL approach is able to better catch the experimental behavior in the central part of the specimen. It is worth noting that the experimental measures become more uncertain when materials and bond behavior attain the nonlinear field.

Moreover, the AL approach presents a cusp at the loaded end of the plate because a flexural effect of the plate is also considered in the model due to the normal stiffness of the adhesive layer. Such an effect is absent in the IE model since the adhesive is modeled by zero-thickness interface elements.

**Figure 7:**
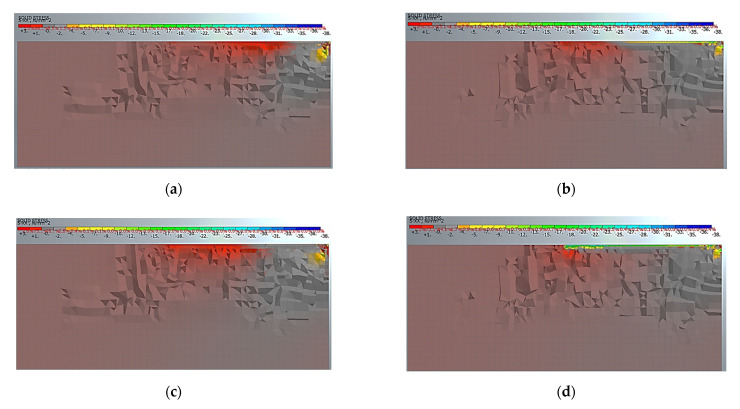
Longitudinal distribution of tensile stresses in the central section of the concrete block for the specimen C30_1.4_50_250 at: (**a**) 0.8 P_u_ for IE model; (**b**) 0.8 P_u_ for AL model; (**c**) at P_u_ for IE model; (**d**) at P_u_ for AL model.

In Figure 7, the longitudinal distributions of the tensile stresses in the concrete at the central section is reported at two load levels for the specimen C30_1.4_50_250. In particular, the tensile stresses in the concrete at 80% of the ultimate load are shown in Figure 7a,b and at failure in Figure 7c,d. The damage distribution in the concrete during the debonding process in the IE and AL models can be appreciated and it can be noted that the damage is widespread along the entire bonded length in both models. In the AL model (Figure 7b,d), it is possible to observe the effect of the adhesive layer, moreover at failure.

Finally, it is worth noting that the evaluation of the slips provided by the AL approach is not reliable, because the slips at the concrete-adhesive and the plate adhesive-interface are not taken in account, while in the IE approach they are directly included in bond laws experimentally calibrated.

### 5.2. Analysis of Bond Behavior by Means of Parametric Analyses

After the validation of the 3D FE model by means of the comparison with experimental results, the model adopting the AL approach has been used for carrying out a parametric analysis with refer to steel plates bonded to a concrete element with the same dimensions of the one considered in the experimental tests of [9], i.e., b_c_ = 200 mm. Different values of bonded length and width of the steel plate were investigated considering the same two concrete strength previously used. In particular, widths b_p_ = 10, 30 and 50 mm and bonded lengths l_b_ = 50, 150, 250, 350 and 400 mm were considered. Two plate thickness, i.e., t_p_ = 2 or 3 mm, and two concrete strength, i.e., f_c_ = 30 and 50 MPa, were assumed. The adhesive thickness was 1 mm in all simulations. Mechanical properties of steel are invariant and are listed in Table 1. In Table 3, the characteristics of the simulated specimens are reported together with the numerical values of the failure load achieved by the AL FE model, P_u_,_num_, and the theoretical values, P_u,th_, provided by the strength model represented by Equation (3) for mean and characteristic values (with subscript m and k) and Equation (6). Finally, in the last two columns, the differences between the numerical results and the theoretical ones are listed too. Note that the specimens are labelled as Cf_c__t_p__b_p__l_b_, being f_c_, t_p_, b_p_, and l_b_ the parameters previously introduced.

**Figure 8:**
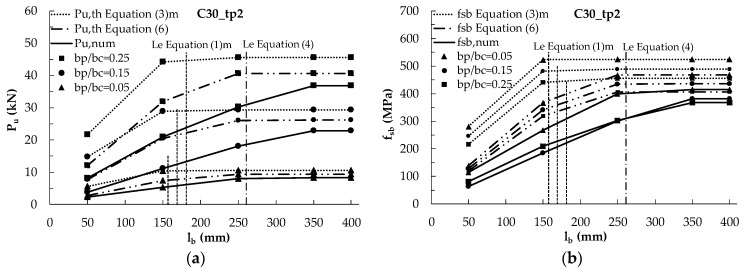

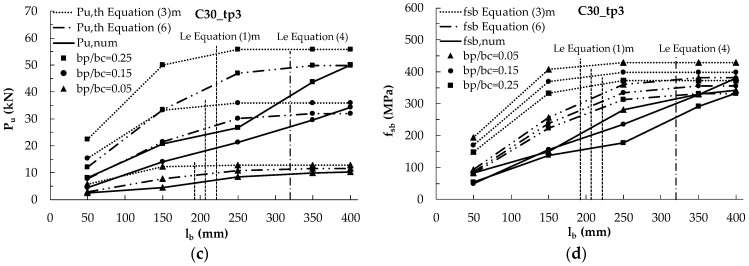
Numerical and mean theoretical debonding load and corresponding tensile stress f_sb_ (Equations (3) and (6)) vs. bonded length for a concrete C30 and different values of b_p_/b_c_ for: (**a**,**b**) t_p_ = 2 mm; (**c**,**d**) t_p_ = 3 mm.

The numerical analyses evidenced that the bonded length clearly affects the debonding load until l_b_ < L_e_. In Figure 8a,c, the variation of the debonding load provided by the AL FE model with the bonded length is plotted for different values of the plate-to-concrete width, b_p_/b_c_, and for two thickness of the plate (2 mm in Figure 8a and 3 mm in Figure 8c) considering a concrete with f_cm_ = 38 MPa. The theoretical values of the debonding load provided by Equations (3) and (6) are also plotted by means of dotted lines. The theoretical predictions are always higher than the numerical ones for l_b_ < 250 mm. 

Moreover, in Figure 8, the dotted vertical lines represent the effective lengths calculated by means of Equations (1) and (4). Equation (1) provides effective lengths variable with the plate-to-concrete width, b_p_/b_c_: for t_p_ = 2 mm they are 160 mm, 170 mm and 180 mm for b_p_/b_c_ = 0.05, 0.15 and 0.25, respectively. For t_p_ = 3 mm, the effective lengths are 190 mm, 200 mm and 220 mm for b_p_/b_c_ = 0.05, 0.15 and 0.25, respectively. Equation (4) furnishes values of the effective length independent from the width and approximately equal to 260 mm and 320 mm for t_p_ = 2 mm and 3 mm, respectively. It is worth noting that the bonded lengths of 350 mm and 400 mm adopted in the FE model are always higher than the theoretical effective length, L_e_, provided by both Equations (1) and (4).

The numerical results show that, for the lowest value of b_p_/b_c_, the effective length is about 250 mm for t_p_ = 2 mm and 300 mm for t_p_ = 3 mm, i.e., more or less equal to the theoretical prediction of Equation (4), while for increasing values of b_p_/b_c_ the numerical effective length increases and the theoretical predictions are not always safe. In general, for whatever value of b_p_/b_c_, the numerical effective length is higher than the theoretical one given by Equation (1) and such a difference is as more relevant as b_p_/b_c_ increases and for thicker plates (see Figure 8c for t_p_ = 3 mm). It is, indeed, evident that the numerical effective lengths depend on the bonded width, b_p_, of the plate, in agreement with the predictions of *fib* Bulletin 90 [27], but more significantly. 

The underestimation of the effective length provided by Equation (1) clearly leads also to the overestimation of the debonding load provided by Equation (3) when l_b_ is lower than the numerical values of L_e_. However, there is an overestimation also when l_b_ is lower than the theoretical values of L_e_ provided by Equations (1) and (4), probably because of a not suitable estimation of the reductive factor β*_L_*. Such a difference is more significant for increasing values of b_p_/b_c_ and of t_p_. Conversely, when the numerical debonding load stabilizes, i.e., l_b_ becomes higher than the numerical values of the effective length, the provisions of Equations (3) and (6) are comparable with the numerical ones.

The same results of Figure 8a,c are reported in terms of ultimate stress f_sb_ (being f_sb_ = P_u_/(b_p_t_p_)) in Figure 8b,d, respectively. With reference to the ultimate stress, it can be noted that the effect of b_p_/b_c_ reduces because its variation is due to the variation of the plate width that proportionally increases the bonded area for the ultimate load P_u_. It is interesting to observe that, as for P_u_, also for f_sb_ in all cases the theoretical formulations are unsafe and Equation (3) for t_p_ = 2 mm even provides values of tensile stress higher than the yielding strength of steel (430 MPa). Clearly, these results would not be acceptable for the design and is related to the fact that the theoretical predictions were calibrated on FRP materials that are surely in the elastic field when the debonding occurs. Conversely, thin steel plates with a small width and an adequate bonded length, or provided of end anchorage devices, could allow the yielding of the material; in this case, the bond behavior will be different from that of FRP materials and, therefore, the field of applicability of the theoretical formulations for steel plates should be limited by the yielding strength of steel.

**Figure 9:**
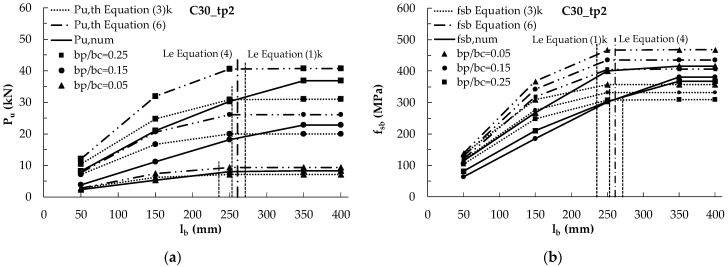

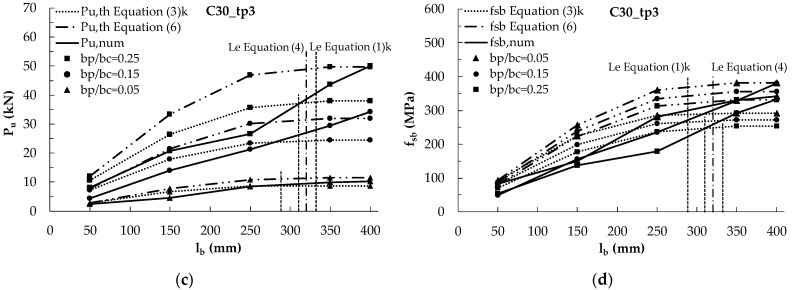
Numerical and characteristic theoretical debonding loads (Equations (3) and (6)) vs. bonded length for a concrete C30 and different values of b_p_/b_c_ for: (**a**) t_p_ = 2 mm; (**c**) t_p_ = 3 mm; ultimate stresses vs. bonded length: (**b**) t_p_ = 2 mm; (**d**) t_p_ = 3 mm.

The characteristic values of the effective length provided by [27] are comparable with the theoretical values provided by the Chen and Teng’s model [16] (260 mm and 320 mm for t_p_ = 2 mm and 3 mm), but also in this case, they are lower than the numerical predictions, as shown in Figure 9. Equation (1) provides, indeed, for t_p_ = 2 mm, characteristic values of the effective lengths equal to about 230 mm, 250 mm and 270 mm for b_p_/b_c_ = 0.05, 0.15 and 0.25, respectively, while, for t_p_ = 3 mm, it provides approximately 290 mm, 310 mm and 330 mm for b_p_/b_c_ = 0.05, 0.15 and 0.25, respectively.

Figure 9 are analogous to Figure 8, with the exception that they compare the numerical results with the theoretical ones provided by the fib model [27], in terms of characteristic values, instead of mean values, for both the ultimate load and the effective length. When the characteristic provisions are used for the *fib* approach, clearly safer values are attained.

**Figure 10:**
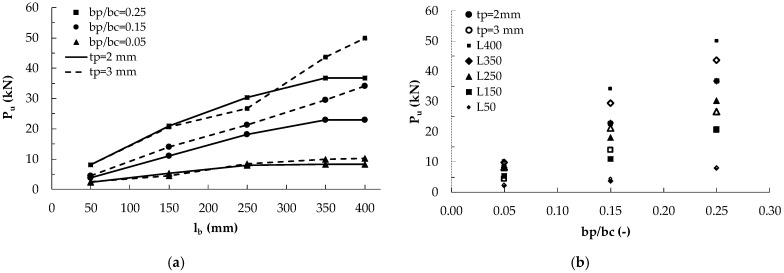
(**a**) Numerical debonding loads vs. bonded length for different values of b_p_/b_c_ and t_p_; (**b**) Numerical debonding loads vs. plate-to-concrete width ratio for different values of l_b_ and t_p_.

The effect of the plate thickness t_p_ and of b_p_/b_c_ on the effective bonded length and the debonding load is evidenced in Figure 10a, where the values of P_u_ provided by the AL FE model are plotted versus the bonded length l_b_ for the two thickness of 2 and 3 mm and different values of b_p_/b_c_. It is worth noting that the ultimate load is strongly dependent on the b_p_/b_c_ ratio because this parameter governs the width of the bonded area. Conversely, the thickness of the plate is influent only for bonded length longer than the effective one (see Figure 10a). Furthermore, greater is t_p_, greater the effective length is.

In Figure 10b, the numerical values P_u_ are plotted versus the plate-to-concrete width ratio for different values of bonded length and plate thickness. Figure 10b shows more evidently the influence of the ratio b_p_/b_c_ on the debonding load. Greater the ratio b_p_/b_c_ is, greater the debonding load is, as previously observed in Figure 10a too, because the width of the bonded area increases with b_p_/b_c_. However, it is worth noting that the increase of the debonding load with b_p_/b_c_ is higher for longer bonded lengths. Therefore, the graphs show the non-linear relationship between P_u_ and b_p_/b_c_, which is not detectable in a 1D or 2D approach.

**Figure 11:**
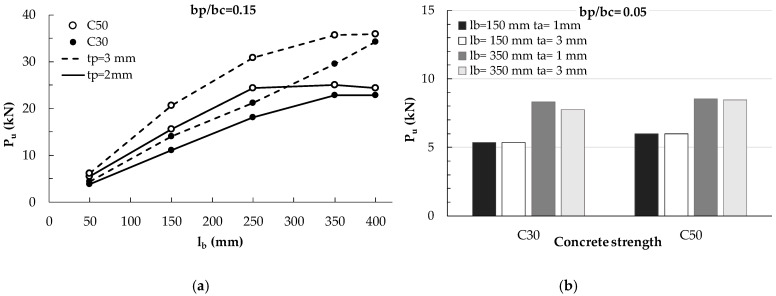
(**a**) Maximum debonding load vs. bonded length for different concrete strength and thickness of steel plate; (**b**) Maximum debonding load for t_p_ = 2 mm by varying bonded length (150 mm and 350 mm), concrete strength (30 and 50 MPa), and adhesive thickness (1 mm and 3 mm).

The effect of concrete strength on the debonding load was investigated too and it is showed in Figure 11a, where P_u_ is plotted versus the bonded length for two values of plate thickness (2 mm and 3 mm) and two concrete strength (30 MPa and 50 MPa) for a selected plate-to-concrete width ratio (b_p_/b_c_ = 0.15). The graphs show that the debonding load increases with the concrete strength mainly for the highest plate thickness (t_p_ = 3 mm), since for higher plate thickness the effective length is higher too and, thus, the debonding load can continues increasing with l_b_. Moreover, it can be noted that the effective length reduces when the concrete strength increases. The numerical results show that the effective length for b_p_/b_c_ = 0.15 and t_p_ = 2 mm is about 250 mm for concrete C30 and about 150 mm for concrete C50, while for t_p_ = 3 mm the effective lengths seem to be longer than 400 mm for C30 and about 350 mm for C50. When the bonded length is greater than the effective one, the ultimate load seems to be insensitive to the substrate strength, but it is worth pointing out that for higher concrete strength a smaller bonded length is required.

Finally, in Figure 11b, the influence of the adhesive thickness (1 mm and 3 mm) on the debonding load is highlighted for a fixed plate thickness (2 mm) and the two concrete strengths, already examined. It can be noted that the influence of adhesive thickness, t_a_, on the debonding load is very low. Within such a limited influence, however, it can be observed that an increase of the adhesive thickness, i.e., a reduction of the adhesive stiffness G_a_/t_a_, leads to a reduction of the debonding load for bonded length longer than effective ones. 

## 6. Conclusions

The paper deals with the analysis of the bond behavior of steel plates externally bonded to concrete elements. A numerical 3D FE model has been developed and, after a validation on experimental results of bond tests available in literature, it has been used for carrying out parametric analysis and comparisons with theoretical formulations in terms of effective length and debonding load. The numerical analyses are useful for assessing the reliability of extending to externally bonded steel plates the design formulations usually adopted for FRP plates. These formulations are well assessed for FRP plates, thanks to the availability of a wide database of experimental results, while the experimental results available for steel plates are very few.

The main results evidenced by the study are:

- The two 3D FE models investigated, i.e., the AL approach that simulates the adhesive with its own thickness and the IE approach that adopts interface elements characterized by shear stress-slip relationships calibrated using experimental tests, give comparable results in terms of effective length and ultimate debonding load; the AL approach has the advantage that allows developing parametric analyses by changing the only properties of materials without necessity of reviewing the bond laws. However, the evaluation of the slips provided by the AL approach is not reliable, because the slips at the concrete-adhesive and the plate adhesive-interface are not computed, while, in the IE approach, they are directly included in bond laws experimentally calibrated.
- The effect of the mesh size on the results provided by the FE model was examined by calibrating the most suitable choice according to the dimension of the strengthening plate (smaller size mesh for smaller plate width), the sensitivity of the results in terms of ultimate load with the mesh size, and the computational effort. A further reduction of the mesh utilized in the analysis should give, indeed, a variation of the results of approximately 2%, but the calculation time would increase significantly.
- The numerical analyses confirm that the bond behavior of externally bonded steel plates is governed by the same parameters influencing that of the FRP plates; in particular, the main parameters are concrete strength, plate axial stiffness, and plate-to-concrete width ratio, but their role depends also on the effective length, which is shorter for higher strength concrete and longer for higher plate stiffness, which is the case of steel plates. The thickness of the adhesive has a little influence on the debonding load.
- About the effect of the axial stiffness of the plate, for bonded lengths lower than the effective ones, the debonding loads seem to be low sensitive to this parameter, while for longer bonded length, they start depending more significantly on the axial stiffness since the latter one influences the effective length too and, thus, the possibility of having bonded length lower or higher than the effective length.

In general, the results herein presented highlight the possibility of considering a unified approach for FRP and steel plates externally glued on concrete elements. The real difference between the performance of FRP and steel plates should be due to the range of variation of the typical axial stiffness of the strengthening plates. The axial stiffness of FRP reinforcements is, indeed, generally lower than that of steel plates, which are used with thickness of millimeters and have a fixed elastic modulus. Conversely, preformed FRP plates have thickness of millimeters, but elastic moduli lower than steel, while wet-lay-up systems may have very high elastic moduli, also higher than steel, but are characterized by very thin thickness. However, the assessment of the effect of thickness and stiffness of the strengthening plates needs of further numerical analyses and comparisons with experimental results.

Moreover, the applicability of the design factors and the details suggested for FRP plates to steel plates probably need to be reviewed because they were calibrated on experimental results and studies using the typical values of stiffness of FRP plates and sheets. Since the experimental results of bond tests on steel plates glued on concrete elements available in literature were few, they were, indeed, not considered in the calibrations used for assessing the current national and international design guidelines for FRP reinforcements.

Therefore, this paper is a preliminary step of a process aimed to assess the bond behavior of externally bonded steel plates. The research will continue with specific experimental bond tests on steel plates aimed to confirm the numerical results herein presented, assess the effect of thickness and stiffness of the strengthening plates, and review the factors present in the design formulations.

## Figures and Tables

**Table 1:** Geometrical and mechanical properties of materials used in the FE.

Materials	f_cm_ (MPa)	f_y_ (MPa)	c (MPa)	ϕ (°)	f_t_ (MPa)	E (MPa)	t (mm)
Concrete C30	38.0	-	1.8	31	2.9	33,000	200
Concrete C50	58.0	-	2.6	31	4.1	37,000	200
Adhesive	80.3	-	-	-	-	500	1.0; 3.0
CFRP	−	-	-	-	2850.0	152,200	1.4
Steel	275.0	275.0	-	-	430.0	210,000	2.0; 3.0

**Table 2:** Comparison between experimental results of [9] and numerical and theoretical debonding loads.

Specimen		C30_1.4_50_250	C30_1.4_50_250
f_c_ (MPa)		30	50
t_p_ (mm)		1.4	1.4
E_p_ (MPa)		152,200	152,200
b_p_ (mm)		50	50
l_b_ (mm)		250	250
P_u,exp_ (kN)		28	34
P_u,th_ (kN)	Equation (3)_m_	32.5	37.4
	Equation (6)	27.3	32.2
P_u_,_num_ (kN)	AL	27.9	35.8
	IE	30.9	34.1
P_u,exp_/P_u,th_ (-)	Equation (3)_m_	0.86	0.91
	Equation (6)	1.03	1.06
P_u,exp_/P_u_,_num_ (-)	AL	1	0.95
	IE	0.91	1
P_u_,_num_^AL^/P_u,th_ (-)	Equation (3)_m_	0.86	0.96
	Equation (6)	1.02	1.11

**Table 3:** Geometries of simulated specimens and maximum debonding loads according to theoretical and numerical models.

Specimen	f_cm_ (MPa)	t_p_ (mm)	E_p_t_p_ (kN/mm)	b_p_ (mm)	l_b_ (mm)	b_p_/b_c_ (-)	P_u,th_ (kN)			P_u,num_ (kN) AL FE	P_u_,_num_/P_u,th_ (-)		
							Equation (3)_m_	Equation (3)_k_	Equation (6)		Equation (3)_m_	Equation (3)_k_	Equation (6)
C30_2_10_50	38	2	420	10	50	0.05	5.6	2.7	2.8	2.3	0.41	0.85	0.83
C30_2_10_150	38	2	420	10	150	0.05	10.5	6.2	7.3	5.4	0.51	0.87	0.73
C30_2_10_250	38	2	420	10	250	0.05	10.5	7.1	9.3	8.0	0.76	1.12	0.86
C30_2_10_350	38	2	420	10	350	0.05	10.5	7.1	9.4	8.3	0.79	1.16	0.89
C30_2_10_400	38	2	420	10	400	0.05	10.5	7.1	9.4	8.3	0.79	1.16	0.89
C30_2_30_50	38	2	420	30	50	0.15	14.8	7.1	7.7	3.8	0.26	0.54	0.49
C30_2_30_150	38	2	420	30	150	0.15	28.9	16.6	20.5	11.1	0.38	0.67	0.54
C30_2_30_250	38	2	420	30	250	0.15	29.3	19.9	26.1	18.1	0.62	0.91	0.69
C30_2_30_350	38	2	420	30	350	0.15	29.3	19.9	26.1	22.9	0.78	1.15	0.87
C30_2_30_400	38	2	420	30	400	0.15	29.3	19.9	26.1	22.9	0.78	1.15	0.87
C30_2_50_50	38	2	420	50	50	0.25	21.7	10.4	12.0	8.1	0.37	0.78	0.67
C30_2_50_150	38	2	420	50	150	0.25	44.2	24.8	31.9	21.0	0.47	0.85	0.66
C30_2_50_250	38	2	420	50	250	0.25	45.6	30.8	40.6	30.3	0.66	0.98	0.75
C30_2_50_350	38	2	420	50	350	0.25	45.6	31.0	40.6	36.8	0.81	1.19	0.91
C30_2_50_400	38	2	420	50	400	0.25	45.6	31.0	40.6	36.8	0.81	1.19	0.91
C30_3_10_50	38	3	630	10	50	0.05	5.8	2.8	2.8	2.5	0.43	0.90	0.90
C30_3_10_150	38	3	630	10	150	0.05	12.2	6.7	7.7	4.5	0.37	0.67	0.58
C30_3_10_250	38	3	630	10	250	0.05	12.9	8.6	10.8	8.4	0.66	0.98	0.78
C30_3_10_350	38	3	630	10	350	0.05	12.9	8.7	11.5	9.9	0.77	1.13	0.86
C30_3_10_400	38	3	630	10	400	0.05	12.9	8.7	11.5	10.3	0.80	1.17	0.90
C30_3_30_50	38	3	630	30	50	0.15	15.3	7.2	7.8	4.4	0.29	0.61	0.57
C30_3_30_150	38	3	630	30	150	0.15	33.2	17.9	21.5	14.0	0.42	0.78	0.65
C30_3_30_250	38	3	630	30	250	0.15	35.9	23.5	30.2	21.2	0.59	0.90	0.70
C30_3_30_350	38	3	630	30	350	0.15	35.9	24.4	32.0	29.5	0.82	1.21	0.92
C30_3_30_400	38	3	630	30	400	0.15	35.9	24.4	32.0	34.2	0.95	1.40	1.07
C30_3_50_50	38	3	630	50	50	0.25	22.3	10.6	12.1	8.1	0.36	0.77	0.67
C30_3_50_150	38	3	630	50	150	0.25	50.0	26.5	33.4	20.7	0.41	0.78	0.62
C30_3_50_250	38	3	630	50	250	0.25	55.8	35.6	46.9	26.7	0.48	0.75	0.57
C30_3_50_350	38	3	630	50	350	0.25	55.8	38.0	49.8	43.7	0.78	1.15	0.88
C30_3_50_400	38	3	630	50	400	0.25	55.8	38.0	49.8	50.0	0.90	1.32	1.00
C50_2_10_150	58	2	420	10	150	0.05	12.1	7.6	8.8	6.0	0.50	0.79	0.68
C50_2_10_350	58	2	420	10	350	0.05	12.1	8.2	10.4	8.5	0.71	1.04	0.82
C50_2_30_50	58	2	420	30	50	0.15	19.1	9.2	9.5	5.5	0.29	0.60	0.58
C50_2_30_150	58	2	420	30	150	0.15	33.7	20.6	24.5	15.6	0.46	0.76	0.64
C50_2_30_250	58	2	420	30	250	0.15	33.7	22.9	29.1	24.4	0.72	1.06	0.84
C50_2_30_350	58	2	420	30	350	0.15	33.7	22.9	29.1	25.0	0.74	1.09	0.86
C50_2_30_400	58	2	420	30	400	0.15	33.7	22.9	29.1	24.4	0.72	1.06	0.84
C50_3_30_50	58	3	630	30	50	0.15	19.8	9.5	9.6	6.1	0.31	0.65	0.64
C50_3_30_150	58	3	630	30	150	0.15	40.2	22.6	26.0	20.6	0.51	0.91	0.79
C50_3_30_250	58	3	630	30	250	0.15	41.3	28.0	34.8	30.9	0.75	1.10	0.89
C50_3_30_350	58	3	630	30	350	0.15	41.3	28.1	35.6	35.7	0.86	1.27	1.00
C50_3_30_400	58	3	630	30	400	0.15	41.3	28.1	35.6	35.9	0.87	1.28	1.01

## Data Availability

The data presented in this study are available on request from the corresponding author.
